# Heterospecific pollen avoidance strategy prevails in the generalized plant–pollinator network on Yongxing Island

**DOI:** 10.1002/ece3.11123

**Published:** 2024-03-04

**Authors:** Xiang‐Ping Wang, Jin‐Chao Cai, Ma‐Yin Tong, Miao‐Miao Shi, Zhong‐Tao Zhao, Shi‐Jin Li, Tie‐Yao Tu

**Affiliations:** ^1^ Key Laboratory of Plant Resources Conservation and Sustainable Utilization, South China Botanical Garden Chinese Academy of Sciences Guangzhou China; ^2^ South China National Botanical Garden Guangzhou China; ^3^ Gannan Normal University Ganzhou China; ^4^ University of Chinese Academy of Sciences Beijing China

**Keywords:** heterospecific pollen avoidance, network structural properties, oceanic island, phenotypic specialization, pollen deposition, pollination network

## Abstract

Heterospecific pollen (HP) deposition varies widely among species in communities, which has been explicated by two adaptation strategies: HP avoidance and HP tolerance. Studies of the plant–pollinator network have uncovered that oceanic island communities are highly generalized and strongly connected. It remains unclear, however, which strategy prevails in such communities. We examined stigma pollen deposition on 29 plant species, and assessed patterns of HP load size and diversity in the Yongxing Island community. We assessed the effects of phenotypic specialization and species‐level network structural properties of plant species on pollen deposition among species. The hypothesis of three accrual patterns of HP within species was tested by illustrating the relationship between conspecific pollen (CP) and HP receipt. Extensive variation occurred among species in HP receipt, while 75.9% of species received less than 10% HP and one species received more than 40% HP throughout the community. Flower size strongly drives the variation of HP receipt, while network structural properties had no effect on the pollen receipt. Nineteen species showed no relationship between the number of HP and CP loads, and they received smaller HP load sizes and lower HP proportions. Most plant species evolved HP avoidance strategy, and HP receipt was an occasional event for most plant species in the generalized community. HP and CP receipts are independent of each other in plant species with the HP avoidance mechanism. Our results highlight that plants in the generalized pollination system may preferentially select to minimize the HP load on stigmas.

## Graphical abstract

Flower size strongly drives the variation of HP receipt, while network structural properties had no effect on the pollen receipt. Most plant species evolved HP avoidance strategy, and HP receipt was an occasional event for most plant species in the generalized community.

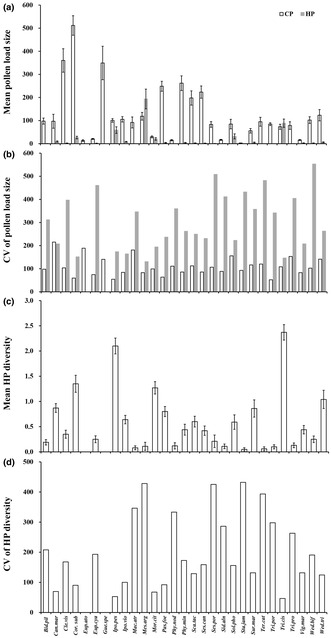

## 1 INTRODUCTION

Oceanic islands usually have poor species and links due to their high isolation from continents, which is a large obstacle to the ongoing arrival of many plant and animal species (Olesen & Jordano, 2002; Traveset et al., 2016; Trøjelsgaard & Olesen, 2013). Thus, small island communities often have a more generalized structure and a higher connectance in plant–pollinator interaction networks when compared with mainland communities, leading to a high pollinator overlap (Traveset et al., 2016; Wang, Wen, Qian, et al., 2020; Wang, Zeng, et al., 2020). This overlap may increase the possibility of heterospecific pollen (HP) transfer and deposition on stigmas (Fang & Huang, 2016; Flanagan et al., 2009; Huang et al., 2015; Traveset et al., 2013). HP deposition on stigmas has negative impacts on plant reproduction through clogging stigmas or pollen interference, which further affects the structure of plant communities (Arceo‐Gómez, 2021; Arceo‐Gómez, Kaczorowski, et al., 2019; Ashman & Arceo‐Gómez, 2013; Briggs et al., 2015; Fang et al., 2023; Muchhala & Thomson, 2012). On oceanic islands, plant and pollinator species tend to be generalized, and some can even be super‐generalists; this means that plants are visited by many pollinator species, and pollinators, in turn, visit a number of plant species (Traveset et al., 2013; Traveset et al., 2016; Wang et al., 2021; Wang, Fu, Shi, Zhao, et al., 2023; Wang, Wen, Qian, et al., 2020; Wang, Zeng, et al., 2020). Such a high pollinator overlap in these oceanic island communities may encourage more common HP transfer, resulting in negative consequences for overall plant fitness.

The proportion of HP deposition on stigmas varies greatly across species, ranging from less than 0.1% to 70% (Ashman & Arceo‐Gómez, 2013; Bartomeus et al., 2008; Fang et al., 2019; Fang & Huang, 2013; Johnson & Ashman, 2018; Montgomery & Rathcke, 2012). Ashman and Arceo‐Gómez (2013) proposed that plant species with a high frequency of HP deposition would either select HP avoidance or tolerance to minimize the deleterious effects, and that these two alternative evolutionary strategies could promote continued pollinator sharing as well as the coexistence of plant species in communities. The HP avoidance mechanism can further decrease the receipt of HP, while the HP tolerance mechanism can constantly accept large numbers of HP (Arceo‐Gómez, 2021; Arceo‐Gómez, Abdala‐Roberts, et al., 2016; Ashman & Arceo‐Gómez, 2013; Hao et al., 2023). Fang et al. (2019) first examined these two mechanisms among plant species in a subalpine community; it was further supported that HP avoidance or tolerance mechanisms have evolved in some species and that variation in HP receipt among plant species is likely the result of different species “properties.” Fang et al. (2019) also indicated, however, that only three species selected the HP tolerance mechanism while 21 species selected the HP avoidance mechanism, raising the question of whether the HP avoidance mechanism is frequent than the HP tolerance mechanism within other communities as well. We speculated that plants would mostly evolve the HP avoidance mechanism to decrease HP receipt, especially in a generalized pollination system, which may be at a greater risk of receiving HP considering how the HP load on stigmas can significantly affect plant reproduction (Arceo‐Gómez, Kaczorowski, et al., 2019; Briggs et al., 2015; Lanuza et al., 2021). Nevertheless, studies exploring the practicability of HP tolerance and avoidance mechanisms of plants in different communities are still scarce, especially those centered around generalized pollination systems.

Plant–pollinator network metrics such as species specialization describe the function of a single species within the community (Blüthgen et al., 2006; Coux et al., 2016; Koski et al., 2015). Plant species interaction strength reflects the pollinator dependencies (Bascompte et al., 2006), which may relate to the variation of pollinator visitation rate. CP deposition would thus increase with increasing plant–species interaction strength. Moreover, plant species specialization directly relates to the number of pollinator species, meaning more specialized plants are visited by a smaller visitor diversity (Blüthgen et al., 2006). This specialization then limits pollinator sharing with other co‐flowering plant species and hence HP transfer. In accordance with this relationship, highly specialized plants can be expected to receive a lower HP load size and HP diversity. Plant species closeness centrality represents the proximity to other pollinator species in the network (Martin‐Gonzalez, 2010); thus, plants with high closeness centrality would experience large HP deposition on stigmas as they tend to interact with more generalized pollinator species. Exploring the relationship between the role of species within networks and the patterns of pollen deposition can help us to better understand the relationship between structural network metrics and ecological function in communities.

Floral traits can affect the interaction between plants and pollinators and, in turn, affect the HP deposition within the community (Ashman & Arceo‐Gómez, 2013; Lanuza et al., 2021; Minnaar et al., 2019). For instance, floral symmetry determined the amount of HP deposition across plant communities worldwide (Arceo‐Gómez, Schroeder, et al., 2019). Specifically, radial flowers are usually visited by more pollinator species and may receive a larger HP load size and diversity compared to bilateral flowers (Arceo‐Gómez, Abdala‐Roberts, et al., 2016; Montgomery & Rathcke, 2012). However, this prediction has not been borne out because studies within single communities have inconclusive results (McLernon et al., 1996; Montgomery & Rathcke, 2012). Hence, the influence of floral symmetry on the HP receipt of plants across different community types is still a topic that warrants further research. On the other hand, plants with large flowers are available to a wide range of pollinator species, while their stigmas are expected to receive abundant and diverse amounts of HP (Arceo‐Gómez, 2021; Ashman & Arceo‐Gómez, 2013; Fang & Huang, 2013; Hao et al., 2023; Montgomery & Rathcke, 2012). There are still few studies that indicate how floral traits determine the impact of CP and HP deposition among species, especially in the generalized pollination systems.

The HP load among individuals within a single species also varies extensively, though this species‐level variation has received little attention (Arceo‐Gómez, Abdala‐Roberts, et al., 2016; Ashman et al., 2020). Arceo‐Gómez, Abdala‐Roberts, et al. (2016) proposed three patterns of HP deposition among plants within species that can be assessed by describing the relationship between CP and HP receipt. The three HP–CP patterns include: (1) Increasing linearly: the HP receipt increases as CP increases when each pollinator visit brings both CP and HP grains; (2) No relationship: HP receipt is independent of CP receipt when only a few high‐quality pollinators bring almost pure CP and an occasional few HP grains; (3) Increasing or decreasing exponentially: HP receipt decreases or increases exponentially as CP receipt increases when both high‐quality and low‐quality pollinators visit flowers simultaneously in a species, respectively. These patterns are based on the premise that the visitation rate of high‐quality pollinators is also higher than that of low‐quality pollinators, which has often been reported in natural communities (Gómez et al., 2007, 2010; Sahli & Conner, 2006). In generalized pollination systems, such as oceanic island communities, visitation patterns may differ. For example, low‐quality pollinators can also visit flowers more frequently than high‐quality pollinators in some plant species in an oceanic island (Wang, Wen, Wu, Xu, et al., 2020; Wang, Wen, Wu, & Zhang, 2020). Furthermore, pollen transfer depends not only on visitation rate (Minnaar et al., 2019) but also on the plant's operation of pollen deposition via floral morphological adaptation (Moreira‐Hernández & Muchhala, 2019). For instance, the relative positioning of anthers and stigmas can mediate the location of pollen on a pollinator's body (Huang et al., 2015; Minnaar et al., 2019; Tong & Huang, 2016), increasing CP movement and reducing HP deposition. Pollen grain morphological characteristics can also influence the ability of pollinators to carry pollen, which can be regarded as another HP avoidance mechanism restricting and influencing HP pollen transport (Konzmann et al., 2019; Minnaar et al., 2019). This plant HP avoidance can impact the visitation rate of pollinators and further affect the CP–HP relationship. Therefore, we hypothesized that when plants evolved the HP avoidance strategy, there would be a non‐significant CP–HP relationship, and when plants evolved the HP tolerance strategy, there would be a significant linear or exponential CP–HP relationship.

In this study, we first verified the network‐level specialization of the whole pollination network and the species‐level specialization of plant and pollinator species within the Yongxing Island community. We evaluated data on the species composition of pollen on stigma to explore which mechanism indicated to be most prevalent within the oceanic island pollination system. We also evaluated the relative importance of structural network properties and floral traits as determinants of differences in variance of HP load size, diversity, and proportion received by flowers among plant species. We tested the predictions of the relationship between CP–HP patterns and tolerance‐avoidance strategies by characterizing CP–HP relationships within species variation in HP deposition. Our study of CP and HP receipt in the oceanic island community of Yongxing Island is the first assessment of the association between CP–HP receipt patterns and HP tolerance‐avoidance strategies, providing us with more rigorous information about the plant–plant interactions and species coexistence.

## 2 MATERIALS AND METHODS

### 2.1 Study site and periods

The Paracel Islands (Xisha Islands) are a series of coral islets located in the South China Sea (15°46′–17°08′ N, 110°11′–112°54′ E). Yongxing Island (16°49′ N, 112°20′ E), with a total area of 2.6 km^2^, is the largest islet of this archipelago (for more details on its location, see Wang, Wen, Qian, et al., 2020). We collected the pollination data for each flowering plant species from July 5, 2018 to August 25, 2018. Plant species were in bloom, while active pollinator species did not change across the 2‐month study period. We collected data at the species level in different locations to maximize the possibility of detecting different pollinator species and reduce the effects of geographical distribution.

### 2.2 Pollination data collection

We conducted 15 5 × 5 m quadrats located across the island and at least 100 m away from one another. Pollinators were investigated on sunny days without wind between 8:30 and 16:00 h during peak pollinator activity. Our observations were conducted in the above 15 quadrats. Visits to the flowers of each plant species were recorded during 30‐min observations in each sampling interval. In total, each plant species in the 15 quadrats was observed for two sunny days. For each observation interval, we only recorded visits where pollinators made contact with the flower's reproductive organs (anther and/or stigma) for more than 2 s. We observed 29 plant species in total within the study period (Table 1). Almost all flower visitor species in the Yongxing Island community have been identified to the species level in our previous studies (Wang et al., 2021; Wang, Fu, Shi, Xue, et al., 2023; Wang, Fu, Shi, Zhao, et al., 2023; Wang, Wen, Qian, et al., 2020; Wang, Zeng, et al., 2020). Thus, the visitation rate of each visitor species to each plant species can be accurately recorded in the field.

**TABLE 1:** Floral symmetry (R: radial symmetry; B: bilateral symmetry), flower size (mm^2^), and heterospecific pollen (HP) proportion of the 29 plant species in the Yongxing Island community.

Species code	Floral symmetry	Formula	Flower size	HP %
Bid.pil	R	πr^2^	488.5 ± 25.5	0.5
Can.mar	B	L × W + l × w	861.3 ± 18.5	11.3
Cle.vis	B	L × W	204.0 ± 6.4	0.9
Cor. sub	R	πr^2^ + πBD	2235.1 ± 71.8	4.1
Eup.ato	R	πr^2^	188.0 ± 11.6	0
Eup.cya	R	πr^2^	2333.9 ± 140.2	3.9
Gue.spe	R	πr^2^ + πBD	1069.4 ± 41.8	0
Ipo.pes	R	πr^2^ + πBD	4454.6 ± 120.8	25.4
Ipo.vio	R	πr^2^ + πBD	9415.9 ± 266.5	6.4
Mac.atr	B	L × W + l × w	427.6 ± 10.1	0.2
Mes.arg	R	πr^2^	22.9 ± 1.6	38.1
Mor.cit	R	πr^2^ + πBD	260.7 ± 5.6	33.3
Pas.foe	R	πr^2^	1073.7 ± 27.2	2.3
Phy.nod	B	πr^2^	4.2 ± 0.2	1.9
Phy.min	R	πr^2^ + πBD	142.1 ± 5.6	1.5
Sca.tac	B	L × W	405.7 ± 25.5	3.3
Ses.can	B	L × W + l × w	147.6 ± 3.1	1.1
Ses.por	R	πr^2^	148.0 ± 5.6	1.9
Sid.aln	R	πr^2^	136.7 ± 6.5	1.6
Sol.pho	R	πr^2^	47.4 ± 2.9	10
Sta.jam	B	L × W + πBD	177.1 ± 2.3	0.6
Sur.mar	R	πr^2^	102.7 ± 1.2	11.6
Ter.cat	R	πr^2^	45.5 ± 3.3	0.6
Tri.por	B	L × W	54.8 ± 4.4	0.2
Tri.cis	R	πr^2^	577.8 ± 40.1	41.8
Tri.pro	R	πr^2^	158.5 ± 5.0	0.5
Vig.mar	B	L × W + l × w	396.7 ± 9.1	10.3
Wed.bif	R	πr^2^	590.5 ± 51.1	1.2
Wed.tri	R	πr^2^	843.5 ± 31.1	4.2

*Note*: The meanings of each formulae in each plant species are shown in our previous study (Wang, Wen, Qian, et al., 2020). “Flower formulae” for the measurement of the area of floral visual units: πr^2^ (*r* = corolla radius) for flowers with circular outlines; *L* × *W* (*L* = corolla length, *W* = corolla width) for bilaterally symmetrical with flat flowers; *L* × *W* + πBD (*L* = flat corolla length, *W* = flat corolla width, *B* = bore diameter, *D* = depth of tubular part) or πr^2^ + πBD (*r* = radius of circular part, *B* = bore diameter, *D* = depth of tubular part) for flowers had both tubular structure and flat corolla; and *L* × *W* + *l* × *w* (*L* = length, *W* = width of banner, *l* = length, *w* = width of wing) for leguminous plants. For complete taxonomic names for plant species, see Table S1.

### 2.3 Measurements of flower traits

We categorized flowers by bilateral and radial symmetry according to the morphological characteristics of corolla or inflorescences (Hegland & Totland, 2005). Flowers with radial symmetry or inflorescences are bowl‐shaped or trumpet perianths with easily accessible pollen or/and nectar. Flowers with bilateral symmetry, however, are more structurally complex, where pollen or/and nectar are hidden in the corollas, so only morphologically matched pollinators can enter. The Asteraceae and Euphorbiaceae were classified inflorescence in this study. The flower size of each plant species has been measured with a digital caliper and calculated according to their flower or inflorescences shape, as described in our previous study (Wang, Wen, Qian, et al., 2020).

### 2.4 Stigma sampling

We sampled 29 flowering species from 16 families widely distributed in the Yongxing Island community. We collected each plant species' stigmas from the above 15 quadrats on the island to reduce the effect of neighborhood context on pollen deposition. We collected stigmas of each plant species from their wilted flowers on sunny days. At least 50 (50–58) stigmas per plant species were sampled and investigated. Every stigma was randomly collected from different individual flowers across the 15 quadrats from all over the island. Of the 29 species studied, we totally collected 1494 stigmas and stored them separately in clean microcentrifuge tubes containing FAA solution (solution of formalin: glacial acetic acid: 70% ethanol = 1:1:18). In the lab, each stigma was softened in 8 mol/L NaOH solution for 3 h and then put on a clean slide. The falling pollen grains from stigmas into the tubes were centrifuged and transferred from the bottom 30 μL to a clean slide. We examined CP and HP grains from each stigma under a light microscope (BX41, Olympus) and identified them according to morphological features such as size and shape. Pollen grains from the 29 plant species can be distinguished based on their shape and size, even in the four Asteraceae species (Wang, Fu, Shi, Zhao, et al., 2023). A pollen library (except wind‐pollinated species) was constructed from all flowering species simultaneously to help in the identification of pollen grains. We recorded the numbers of CP and HP as well as the number of species of HP on each stigma. To evaluate the among‐species dynamic of pollen deposition, the mean and coefficient of variation (CV) of HP load size, that is, the number of HP, and diversity, that is, the number of HP species, were calculated for each species. The CV (SD/mean) was calculated by treating each stigma as one estimate of HP and CP. The proportion of HP was calculated according to the formula HP/(CP + HP) × 100%. The average number of stigmas observed per species was 51.5 ± 0.3 (mean ± SE). Among all examined stigmas, 84 (5.6%) received no CP and HP pollen grains, so we excluded them from our analysis.

### 2.5 Data analysis

Here, we constructed one qualitative plant–pollinator interaction network. The network was bipartite, with links between plant species and pollinator species. We calculated one network‐level metric and three species‐level metrics (Arceo‐Gómez et al., 2020; Lucas et al., 2018). Network‐level specialization (H_2_′) describes the whole level of specialization of all species in the network (Blüthgen et al., 2006). Species‐level specialization (d') describes the level of specialization of each species, with higher values indicating higher specialization (Blüthgen et al., 2006). Species strength quantifies the sum of the dependencies of each species (Bascompte et al., 2006). Closeness centrality describes the centrality of a species in the network by its path lengths to other nodes (Martin‐Gonzalez, 2010). We estimated the sampling completeness for the constructed plant–pollinator network and computed the Chao 1 estimator of species richness using the iNEXT package (Hsieh et al., 2014) in R. All the network metrics were analyzed using the “bipartite” package version 2.05 in R3.4.4.

Means and CVs of CP load size, means and CVs of HP load size, HP diversity, and HP proportion were calculated for each plant species. These calculations were then used in among‐species regressions to determine the relative importance of the above network metrics and floral traits in explaining interspecific variation in CP and HP deposition. Bilateral and radial symmetry were defined as the numbers 1 and 2, respectively. We calculated standardized regression coefficients for each variable to facilitate comparisons among independent variables. Flower size, HP proportion (log10 transformed), plant specialization (d') (square root), species strength (square root), and closeness centrality (square root) were transformed to improve the distribution of residuals. Data analysis was conducted using IBM SPSS 19.0.

Following Arceo‐Gómez, Abdala‐Roberts, et al. (2016), we examined the relationships between HP and CP pollen loads within species. For each plant species, linear and nonlinear regressions between the number of CP and HP pollen loads per stigma were performed separately. We first tested for the significance of both linear and nonlinear relationships between the CP and HP pollen loads. If we only found a significant linear relationship in the species, then it would be classified as pattern one (increasing linearly). If neither is significant, then the species was classified as pattern two (no relationship). If we found that the nonlinear relationship was significant, then we made an exponential model to fit the data, and this species was categorized as pattern three (increasing or decreasing exponentially). If we found significant linear and nonlinear relationships simultaneously, we selected the model with the best fit by comparing adjusted *R* ^2^s. We then performed linear models to assess whether species with different patterns of CP–HP load also differed significantly in HP load size and diversity, CV of HP load size and diversity, or HP proportion. A one‐way analysis of variance (ANOVA) was used to detect the difference in HP load size and HP proportion between species with a non‐significant CP–HP relationship and species with a significant linear or exponential CP–HP relationship.

## 3 RESULTS

**FIGURE 1:**
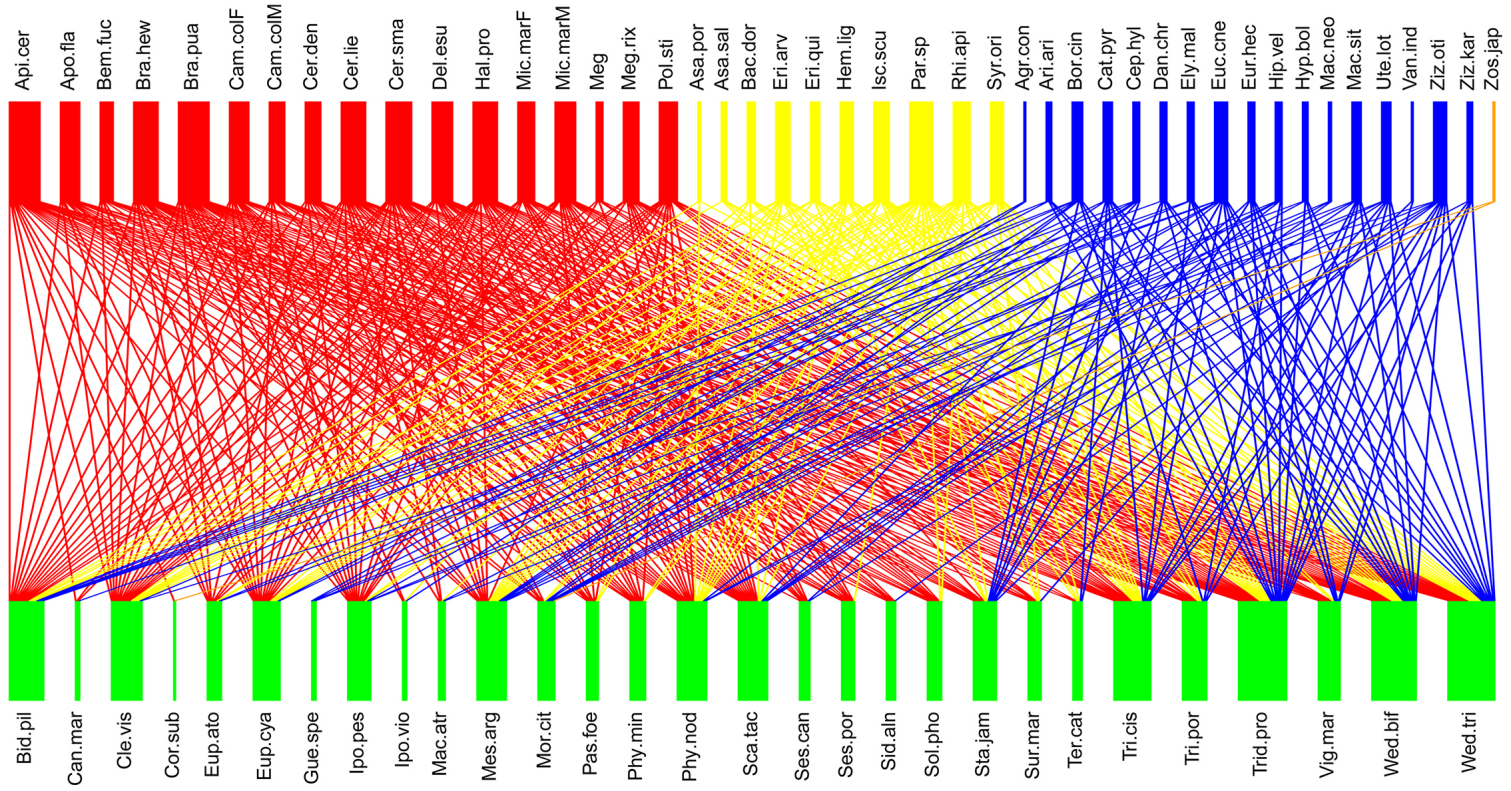
The qualitative plant–pollinator interaction network in the Yongxing Island community. The rectangles represent pollinators (top row) and plant species (bottom row), and the connecting triangles represent links between pollinators and plant species. Pollinator species are color‐coded as follows: red represents Hymenoptera; yellow represents Diptera; blue represents Lepidoptera; and orange represents Passeriformes. Plant species strength, specialization (d'), and closeness centrality were calculated for this network and used to evaluate effects on patterns of pollen receipt. The full names of plant and pollinator species are shown in Table S1.

We totally recorded 494 interactions between 29 plant species and 45 pollinator species during our study periods. The plant species belonged to 16 families, while the pollinator species belonged to four orders (Table S1). Sampling completeness (%) in the plant–pollinator network is 100% (Observed = 29, Estimator = 29, Est_SE = 0.41, 95% Lower = 29, 95% Upper = 30.005). The pollination network was generalized (network‐level specialization: H_2_′ = 0.199) with an average of 6.7 links per species in the network. The values of the species‐level metric d' in both plant and pollinator species were very low (Table S1). Notably, *d*' values of plant species (means ± SE; 0.096 ± 0.021) ranged from 0.007 to 0.491, and the number of interactions for plant species (17.03 ± 1.96) ranged from 2 to 39; *d*' values of pollinator species (0.084 ± 0.018) ranged from 0 to 0.620, and the number of interactions for plant species (10.98 ± 0.925) ranged from 2 to 25 (Figure 1). Wide and small variations were observed in plant species strength (means ± SE; 1.552 ± 0.205; ranged from 0.229 to 4.349) and closeness centrality (0.034 ± 0.0003; ranged from 0.025 to 0.035), respectively (Table S1).

**FIGURE 2:**
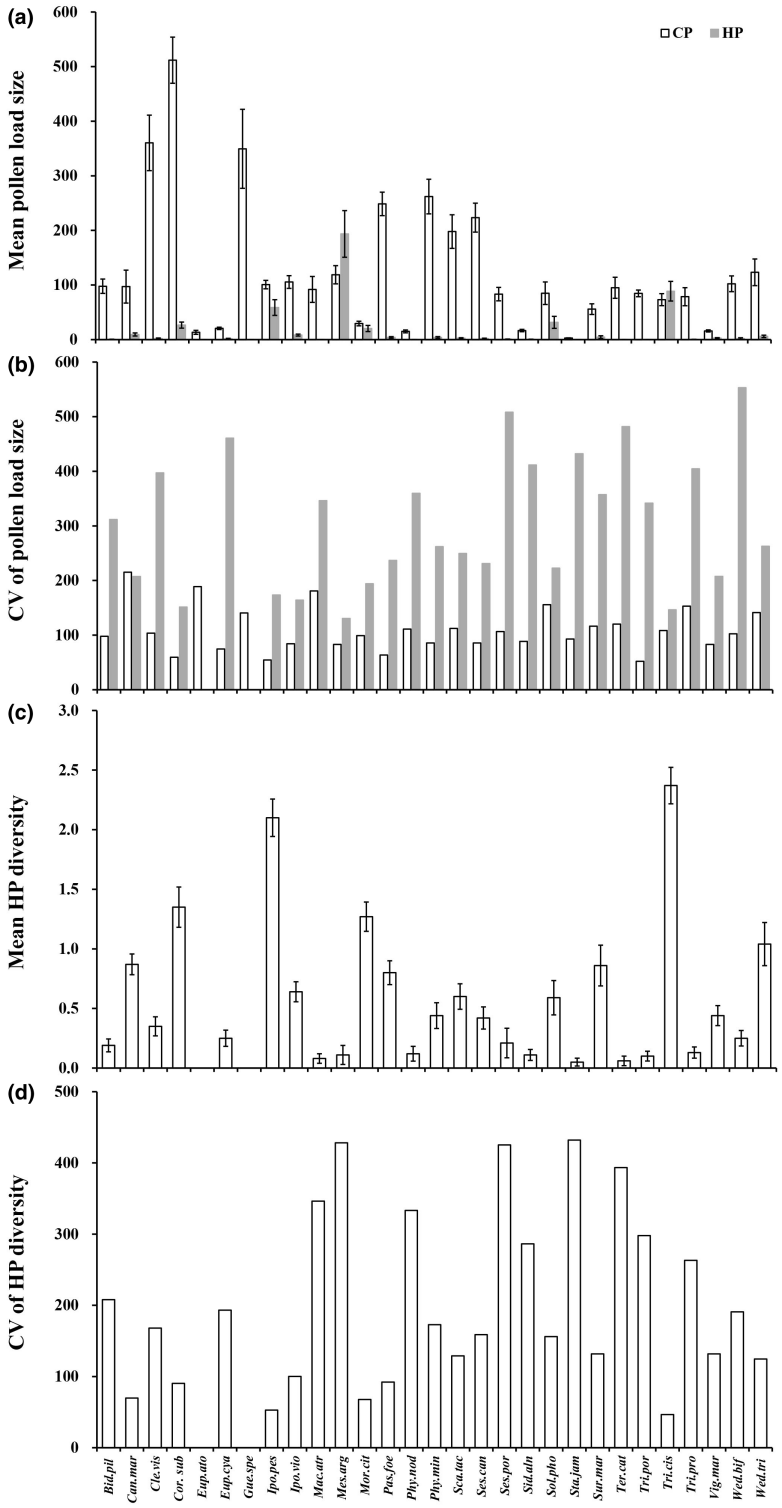
(a) Mean (±SE) for conspecific and heterospecific pollen (CP and HP) load size (number of pollen grains per stigma), (b) Coefficient of variation (CV) for CP and HP pollen load size, (c) Mean (±SE) for heterospecific pollen diversity (number of HP species per stigma), (d) Coefficient of variation (CV) for HP diversity for each of the 29 plant species. Plant species codes follow names in Table S1.

We examined 1494 stigmas in total, counting 203,865 pollen grains, of which 183,244 were conspecific (89.9%) and 20,621 were heterospecific (10.1%). A total of 36.9% of stigmas received heterospecific pollen grains, ranging from 0 to 49 stigmas among these species. We recorded extensive variation among species in mean CP (mean ± SE: 126.2 ± 22.4; range from 2.8 to 511.7 pollen grains) and HP (16.2 ± 7.3; range from 0 to 193.5 pollen grains) load size and HP diversity (0.55 ± 0.11; range from 0 to 2.4 species) per stigma as well as in their respective CVs (109.0 ± 7.5, range from 52.1 to 215.3; 282.9 ± 26.2, range from 0 to 553.1; 189.3 ± 24.1, from 0 to 432.0 for CP load size, HP load size, and HP diversity, respectively; Figure 2, Table S2). Twenty species had flowers with radial symmetry, and nine species had flowers with bilateral symmetry across the studied plant species. There was substantial variation in flower size among species, ranging from 4.21 ± 0.15 to 9415.90 ± 266.49 (Table 1). Among the 29 species, the mean HP proportion (mean ± SE: 7.5 ± 2.2%) was low, ranging from 0% to 41.8% (Table 1). Moreover, 75.9% of species received less than 10% HP, but *Tribulus cistoides* received more than 40% (41.8%) HP.

**FIGURE 3:**
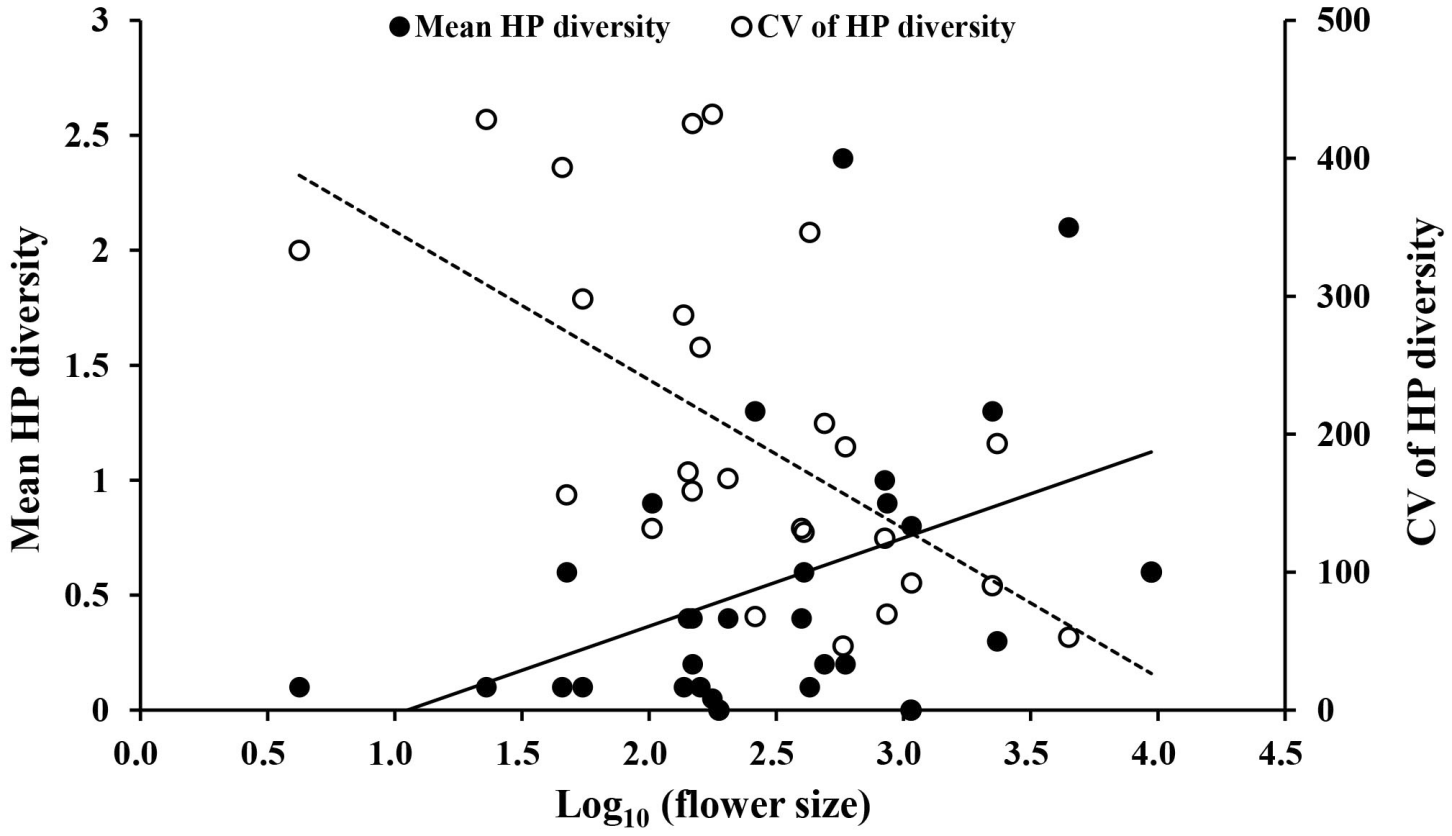
Bivariate plot showing relationships between flower size and mean HP diversity (closed circles, solid line) and CV of HP diversity (open circles, dashed lines) among 29 plant species.

Flower size was the only significant factor that affected HP diversity and CV of HP diversity (Table 2). The mean HP diversity increased significantly with increasing flower size, and the CV of HP diversity reduced significantly with increasing flower size (Table 2, Figure 3). Floral symmetry, plant specialization, species strength, and closeness centrality did not significantly affect the mean and CV of CP load size, the mean CV of HP load size and HP diversity, or HP proportion (Table 2).

**TABLE 2:** Multiple regressions for the effects of floral symmetry, flower size, plant specialization, species strength, and closeness centrality on the mean and variance of HP load size (amount of pollen grains), CP load size, HP diversity (number of pollen species), and HP proportion.

Variables		*b*	*t*	*p*	Model	
Dependent	Independent				*R* ^2^	*p*
Mean HP load size	Floral symmetry	−31.30	−1.84	.08	.21	.46
	Flower size	−14.36	−1.22	.24		
	Plant specialization	289.42	1.58	.13		
	Species strength	−194.06	−1.28	.21		
	Closeness centrality	1913.04	1.04	.31		
CV of HP load size	Floral symmetry	26.76	0.46	.65	.26	.29
	Flower size	−18.76	−0.46	.65		
	Plant specialization	−255.43	−0.40	.69		
	Species strength	−26.54	−0.05	.96		
	Closeness centrality	4273.90	0.68	.51		
Mean CP load size	Floral symmetry	−5.46	−0.11	.91	.33	.14
	Flower size	21.75	0.66	.52		
	Plant specialization	843.03	1.63	.12		
	Species strength	−689.00	−1.62	.12		
	Closeness centrality	−5710.17	−1.11	.28		
CV of CP load size	Floral symmetry	9.61	0.53	.61	.12	.80
	Flower size	−5.23	−0.41	.68		
	Plant specialization	−55.78	−0.28	.78		
	Species strength	−44.65	−0.27	.79		
	Closeness centrality	−2559.29	−1.30	.21		
Mean HP diversity	Floral symmetry	−0.19	−0.76	.46	.30	.21
	Flower size	0.40	2.33	**.03**		
	Plant specialization	0.28	0.11	.92		
	Species strength	0.24	0.11	.91		
	Closeness centrality	36.40	1.37	.19		
CV of HP diversity	Floral symmetry	6.82	0.14	.89	.40	.06
	Flower size	−103.54	−3.06	**.01**		
	Plant specialization	452.91	0.86	.40		
	Species strength	−330.57	−0.76	.46		
	Closeness centrality	6158.46	1.17	.26		
HP proportion	Floral symmetry	−0.37	−1.28	.21	.17	.62
	Flower size	0.15	0.76	.45		
	Plant specialization	−1.31	−0.42	.68		
	Species strength	1.69	0.66	.52		
	Closeness centrality	29.72	0.95	.35		

*Note*: The coefficient of determination (*R* ^2^) and significance (*p*) are shown for each model, along with the standardized regression coefficients (*b*) for each independent variable. Significant models and regression coefficients (*p* < .05) are denoted in bold.

Among the 29 plant species, six plant species (three radial and three bilateral flowers) showed significant linearly increasing relationships between the number of HP and CP depositions, and four species (radial symmetry flowers) showed exponentially increasing relationships (Table 3). Nineteen species showed no pattern between the number of HP and CP loads, that is, HP varies independently of CP (two species received no HP grains; Table 3). Furthermore, differences in HP–CP patterns (among three types) reflected a significant difference in HP diversity (*F* = 20.60, *p* < .001) and flower size (*F* = 3.57, *p* = .043; Table 4). Notably, flowers with an exponential pattern showed the highest values of HP diversity and flower size among the three patterns. Although these relationships were not statistically significant for HP load size, CV of HP load size and HP diversity, floral visitor diversity, or HP proportion, their mean values differed among the three patterns (Table 4). Specifically, plants with an exponential pattern had higher values of HP load size, floral visitor diversity, and HP proportion, but lower values of CV of HP load size and CV of HP diversity. The CVs of HP load size were greater than those of CP in 26 plant species, suggesting that HP receipt generally has higher variation than CP receipt. ANOVA results suggested that there were no significant differences in HP load size (*F* = 0.913, *p* = .348) and HP proportion (*F* = 0.972, *p* = .333) between two types of species, although species with a significant CP–HP relationship have a higher HP load size (means ± SE; 155.7 ± 49.1 vs. 110.6 ± 22.7) and HP proportion (10.5 ± 4.2 vs. 6.0 ± 2.5) than those of species with a non‐significant CP–HP relationship.

**TABLE 3:** The type of HP–CP relationship observed in the 29 plant species in the Yongxing Island community.

Species code	HP–CP pattern	*R* ^2^	*b*	*F*	*p*
Bid.pil (54)	Increasing linearly	.221	0.008	15.71	<.001
Can.mar (50)	Increasing linearly	.388	0.142	22.52	<.001
Cle.vis (54)	Increasing linearly	.511	0.042	16.66	.001
Cor. Sub (52)	Increasing exponential	.364	0.003	19.92	<.001
Eup.ato (50)	No pattern	/	/	/	/
Eup.cya (51)	No pattern	−.074	−0.012	0.24	.636
Gue.spe (51)	No pattern	/	/	/	/
Ipo.pes (50)	Increasing exponential	.175	0.012	10.53	.002
Ipo.vio (58)	Increasing linearly	.08	0.048	5.94	.018
Mac.atr (52)	No pattern	−.021	0.00001	0.00	.962
Mes.arg (50)	No pattern	−.041	0.13	0.06	.803
Mor.cit (50)	No pattern	.022	0.01	1.89	.177
Pas.foe (54)	No pattern	−.01	0.006	0.47	.494
Phy.min (50)	No pattern	.036	0.011	2.85	.098
Phy.nod (51)	No pattern	−.024	0.002	0.08	.778
Sca.tac (52)	No pattern	−.015	0.002	0.26	.614
Ses.can (52)	No pattern	.005	0.004	1.26	.267
Ses.por (52)	No pattern	.002	−0.005	1.13	.293
Sid.aln (50)	No pattern	−.006	0.017	0.71	.404
Sol.pho (50)	Increasing linearly	.606	0.417	22.52	<.001
Sta.jam (50)	Increasing linearly	.145	0.034	7.96	.007
Sur.mar (50)	No pattern	−.039	−0.002	0.26	.617
Ter.cat (50)	No pattern	−.03	0.00007	0.01	.928
Tri.cis (52)	Increasing exponential	.092	0.007	5.84	.02
Tri.por (52)	No pattern	−.011	0.001	0.43	.517
Tri.pro (54)	No pattern	−.017	0.001	0.11	.746
Vig.mar (50)	No pattern	−.053	0.035	0.10	.754
Wed.bif (53)	No pattern	−.018	0.004	0.07	.788
Wed.tri (51)	Increasing exponential	.367	0.004	18.42	<.001

*Note*: The number of stigmas used per species is indicated in parentheses after the species name. For each HP–CP regression, the adjusted *R* ^2^s, the coefficient (*b*), and the significance are shown. A slash represents data not available. For complete taxonomic names for plant species, see Table S1.

**TABLE 4:** Comparison of HP load size, CV of HP load size, HP diversity, CV of HP diversity, floral visitor diversity, HP proportion, plant specialization, and flower size among three CP–HP patterns.

CP–HP pattern	Linearly	Exponential	No pattern	*F*	*p*
	Mean				
HP load size	8.63 ± 4.87	44.95 ± 18.16	12.59 ± 10.11	1.28	.3
CV of HP load size	289.10 ± 44.47	183.45 ± 27.06	301.90 ± 35.93	1.19	.32
HP diversity	0.46 ± 0.13	1.70 ± 0.33	0.33 ± 0.08	20.6	**<.01**
CV of HP diversity	188.98 ± 52.61	78.55 ± 18.13	212.72 ± 30.75	1.88	.17
Floral visitor diversity	15.33 ± 4.22	22.25 ± 7.79	16.47 ± 2.24	0.58	.57
HP proportion	0.38 ± 0.26	1.06 ± 0.26	0.30 ± 0.15	2.45	.11
Plant specialization	0.27 ± 0.06	0.30 ± 0.11	0.27 ± 0.03	0.06	.94
Flower size	2.64 ± 0.32	3.17 ± 0.20	2.26 ± 0.15	3.57	**.04**

*Note*: Significant differences (*p* < .05) are denoted in bold. Mean values (±SE) are given.

## 4 DISCUSSION

Our results of network analysis showed that the whole plant–pollinator interaction network is generalized. These results support previous findings that plants and pollinators tend to be more generalized on oceanic islands than on the mainland and that some plant species can even be super‐generalists (Traveset et al., 2013; Traveset et al., 2016; Wang et al., 2021; Wang, Fu, Shi, Zhao, et al., 2023; Wang, Wen, Qian, et al., 2020; Wang, Wen, Wu, Xu, et al., 2020; Wang, Zeng, et al., 2020). An example would be *Opuntia* spp., which is present in the Galápagos (Traveset et al., 2013) and the Canary Islands (Padrón et al., 2009). In our study, two pollinator species, *Apis cerana* and *Braunapis puangensis*, were two super‐generalist pollinators in the community, which visited 25 plant species, respectively. Highly generalized plant–pollinator networks and species may lead to high HP transfer among co‐flowering plant species in the Yongxing Island community.

Our study of pollen deposition in 29 plant species suggested that HP proportion, mean, and CV of CP and HP load size and diversity varied significantly among plant species. Similar large differences have been found among species within various communities, including alpine (Fang et al., 2019; Fang & Huang, 2013), prairie (Montgomery & Rathcke, 2012), midsuccessional old field (McLernon et al., 1996), dolomite outcrops, and dry scrublands (Arceo‐Gómez, Abdala‐Roberts, et al., 2016). Previous studies reported that ca. 60% of species are low HP receivers (received less than 10% HP), while less than 10% of species are high HP receivers (had over 50% HP) in meadow communities (Fang et al., 2019; Fang & Huang, 2013). In the Yongxing Island community, 75.9% of species received less than 10% HP, but only one species (*Tribulus cistoides*) received more than 40% HP, suggesting that most plant species just experienced interspecific pollen transfer occasionally. Although HP avoidance and HP tolerance are both evolutionary stable strategies for species coexistence (Fang et al., 2019), most species evolved the HP avoidance mechanism for reducing HP deposition on stigmas, especially in a generalized pollination system, which have high pollinator sharing. Generalized plants are more likely to receive HP transferred by generalized pollinators from many other co‐flowering plant species (Arceo‐Gómez, Abdala‐Roberts, et al., 2016; Fang et al., 2023; Lopes et al., 2022). However, although HP deposition on stigmas is mainly carried out by pollinators' moves among co‐flowering plant species, the number of HP grains on stigmas is independent of the number of pollinators' moves. Moreover, plants that receive frequent visits from pollinators that move among other plants can instead maintain lower HP deposition (Fang & Huang, 2016). Supportively, in generalized plant–pollinator networks, most plants may receive lower HP loads and tend to have an HP avoidance strategy. It may be more readily available for plants to evolve the HP avoidance mechanism. For example, the HP avoidance strategy of plants could be achievable by depositing HP on other organs of flowers, such as corollas, instead of stigmas (Murcia & Feinsinger, 1996). Interspecific separation of the sites of pollen assignment and stigma contact on the pollinator body can also reduce the HP load on the stigmas (Huang et al., 2015; Huang & Shi, 2013; Johnson, 2010). Co‐flowering plants that share pollinators can reduce the possibility of HP transfer by stratifying pollen availability to pollinators during the daytime (Štenc et al., 2023). In the Yongxing Island community, the half‐day staggered flowering between morning and afternoon might reduce the number of co‐flowering plant species and thus decrease the probability of HP transfer (Wang et al., 2021). These mechanisms between interacting flowers and pollinators can reduce HP load on stigmas effectively, while the HP tolerance mechanism may often evolve in populations with histories of considerable interspecific pollination (Arceo‐Gómez, Raguso, et al., 2016; Fang et al., 2019; Hao et al., 2023). HP deposition may also affect plant reproductive success; for instance, it can reduce seed set by an average of 20% and even lead to complete reproductive failure (Ashman & Arceo‐Gómez, 2013; Lanuza et al., 2021; Morales & Traveset, 2008; but see Lopes et al., 2022), even though we did not measure the reproductive fitness of plant species here. Therefore, it may be more beneficial for plants to evolve the HP avoidance mechanism rather than the HP tolerance mechanism in natural communities.

Our results revealed that mean HP diversity increased significantly with increasing flower size, and CV of HP diversity reduced significantly with increasing flower size. The HP is more variable than the CP as it is expected to depend on previous visitations on other plants, and not flowers of the same plant. Arceo‐Gómez, Abdala‐Roberts, et al. (2016) found that stigmas from larger flowers received more diverse HP than stigmas from smaller flowers. The effect of flower size on HP pollen deposition may be mediated by enhancing visitation rates (Conner & Rush, 1996) or by larger/more exposed stigmas (Fang & Huang, 2013; Hao et al., 2023; Lanuza et al., 2021; Lopes et al., 2022; Montgomery & Rathcke, 2012). Larger flowers usually have larger stigmas and are thus suitable for visitors of different sizes and can contact more parts of a pollinator's body. This increased contact could increase HP diversity on stigmas, and may be particularly disadvantageous for plants if arriving pollinators bring less proportion of conspecific pollen to stigmas. In addition, in our study, the increase in HP diversity with increasing flower size occurred irrespective of floral visitor diversity, which supports the view that phenotypic specialization does not always reflect ecological specialization (Arceo‐Gómez, Abdala‐Roberts, et al., 2016). The CV of HP diversity reduced significantly with increasing flower size, that is, the CV of HP diversity reduced with increasing HP diversity. This result indicated the similar HP diversity among individuals of species with high HP diversity. The CV of HP diversity increased, as reduced HP diversity may be due to several events of HP deposition in some stigmas, while most other stigmas received no HP. This could be a random “mistake” by pollinators (Fang et al., 2019). In our community, floral symmetry did not significantly affect the HP load size or HP diversity, which is consistent with the study in dolomite outcrops and dry scrublands communities (Arceo‐Gómez, Abdala‐Roberts, et al., 2016) but is contradictory to the results of Arceo‐Gómez, Schroeder, et al. (2019). It is worth noting that the HP receipt rates between flowers with radial and bilateral symmetry diminish with decreasing elevation (Arceo‐Gómez, Schroeder, et al., 2019), suggesting that floral symmetry may only be an effective predictor of HP deposition in plant communities at high elevations, but not in the oceanic island communities. Our results showed that species‐level plant specialization, species strength, and closeness centrality did not significantly affect HP deposition. In grassland communities, however, plant specialization had a significant positive effect, closeness centrality had a significant negative effect, and interaction strength had no effect on HP load size (Arceo‐Gómez et al., 2020). Species‐level network metrics may offer information on the processes influencing patterns of pollen deposition on stigmas for the nested pollination network rather than the generalized pollination network, which was observed here. Network metrics may accurately reflect differences in patterns of pollinator visitation, but they cannot reflect patterns of pollen deposition well (Arceo‐Gómez et al., 2020; King, 2013).

Throughout the 29 plant species in this study, more than 60% showed no significant relationship between the numbers of HP decreasing and CP load. The lack of this relationship suggests that even though most plant species attracted many pollinator species, there were very few effective, high‐quality pollinators that bring relatively pure and large numbers of CP to stigmas. Nevertheless, in several communities, linear and exponentially decreasing patterns of CP–HP relationships prevail (Arceo‐Gómez, Abdala‐Roberts, et al., 2016). This linear CP–HP relationship has been questioned based on the fact that numerous factors would influence this relationship (Ashman et al., 2020). If the visits to plants from all pollinators are even and their visitation effectiveness is similar, there would be linear patterns shown of CP–HP receipt even though the plants' flowers did not evolve the HP avoidance strategy (Arceo‐Gómez, Abdala‐Roberts, et al., 2016; Ashman et al., 2020). Our findings suggested that there will be a high CV of HP load size and low HP diversity in species with exponential patterns of CP–HP receipt due to the number of pollen species deposited during infrequent visits by low‐quality pollinators, which is expected to be lower than that of generalist pollinators with regular and consistent transfer of both CP and HP (Arceo‐Gómez, Abdala‐Roberts, et al., 2016). Thus, not only the quantity but also the quality of pollinators affects CP–HP relationships. This prediction is based on a phenomenon often observed in natural communities that the visitation rate of high‐quality pollinators is often also higher than that of low‐quality pollinators. In increasing exponential patterns of CP–HP receipt, many high‐quality pollinators bring more abundant and purer CP to stigmas, while infrequent low‐quality pollinators bring more various HP to stigmas (Arceo‐Gómez, Abdala‐Roberts, et al., 2016). Contrary to the above hypothesis, four species that exhibited an increasing exponential pattern in our study showed the highest values of HP diversity, HP load size, and HP proportion. In the more generalized networks within oceanic island communities, the low‐quality pollinators could visit flowers more frequently and consistently than the high‐quality pollinators, while the low‐quality pollinators deliver relatively large HP pollen loads. For example, in *Cordia subcordata* (with an exponentially increasing pattern), the low‐quality pollinator, *Apis cerana*, visits flowers more frequently and consistently than the high‐quality pollinator, *Zosterops japonicus*, and *Apis cerana*, which can deposit more HP than CP on stigmas (Wang, Wen, Wu, & Zhang, 2020). A similar case may occur in the other three plant species, though we did not observe the visitation rate and examine the stigmas. Species with significant CP–HP relationships (linear and exponential patterns) have higher HP load size and HP proportion than those of species with non‐significant CP–HP relationships (no pattern), although these differences were not statistically significant. This result supported our prediction that non‐significant CP–HP relationships would be present in species with the HP avoidance strategy, and significant linearly or exponentially CP–HP relationships would be in plants with the HP tolerance strategy. A recent study also found positive CP–HP relationships in two *Silene* species which with HP tolerance strategy (Hao et al., 2023). Our results revealed a pattern suggesting that floral avoidance is effective and excludes most HP transfer in a generalized pollination network, resulting in a non‐significant CP–HP relationship with slight variation in HP load among plant species. More species‐level research is needed to effectively explore the pattern of CP–HP relationships in more types of communities.

This study investigated the pattern of pollen load in 29 plant species in an oceanic island community. Our findings showed that most species evolved the HP avoidance mechanism in order to minimize the possible deleterious effects of HP deposition within the generalized plant–pollinator network. Furthermore, our results highlight that flower size is a vital determinant of interspecific variation in HP diversity, and that plant–pollinator network structural properties have no effect on pollen deposition. We also illustrated the variation of HP receipt within plant species, suggesting that HP and CP depositions are independent of each other within most plant species at the intraspecific level. Such different CP–HP relationships in the same community reflect may provide the raw material for future evolution to shape HP avoidance or tolerance mechanism. Studies of sigma pollen deposition could provide an important tool for describing community‐level plant–pollinator interactions. To fully understand pollinator‐mediated plant–plant interactions, further studies that combine CP–HP patterns and the reproductive biology of plants, such as floral HP avoidance strategies, are required.

## AUTHOR CONTRIBUTIONS

**Xiang‐Ping Wang:** Conceptualization (equal); data curation (equal); formal analysis (equal); funding acquisition (equal); investigation (equal); methodology (equal); visualization (equal); writing – original draft (equal); writing – review and editing (equal). **Jin‐Chao Cai:** Data curation (equal); investigation (equal). **Ma‐Yin Tong:** Investigation (equal); methodology (equal). **Miao‐Miao Shi:** Data curation (equal); methodology (equal). **Zhong‐Tao Zhao:** Methodology (equal). **Shi‐Jin Li:** Methodology (equal). **Tie‐Yao Tu:** Conceptualization (equal); funding acquisition (equal); methodology (equal); writing – review and editing (equal).

## CONFLICT OF INTEREST STATEMENT

All authors declare no conflict of interest and gave final approval for publication.

## Supporting information


Table S1.

Table S2.

## Data Availability

All supplementary data needed to evaluate the conclusions of the paper are in the paper and/or supplementary materials (Tables S1 and S2). Additional data relevant to this paper that support the findings of this study are available from the corresponding author upon reasonable request.
